# Singling out how genes are regulated during development

**DOI:** 10.1016/j.xgen.2021.100087

**Published:** 2022-01-13

**Authors:** Shawn M. Burgess

**Affiliations:** 1 Translational and Functional Genomics Branch, National Human Genome Research Institute (NHGRI), National Institutes of Health (NIH), Bethesda, MD, USA

## Abstract

In this issue of *Cell Genomics*, McGarvey et al. characterize chromatin accessibility and gene regulation at single-cell resolution in the zebrafish embryo 24 h after fertilization, providing a valuable resource. Their findings add another important piece to understanding the dynamic landscape of gene expression and regulation during early vertebrate development and hint at the dramatic changes single-cell genomics is bringing to the field of developmental biology.

## Main text

In the context of developmental biology, gene expression atlases can provide insights into key developmental stages of an organism. The more we understand how cell fates move down developmental pathways based on the combinatorial activation of genes, the better able we become to manipulate these pathways. Although single-cell expression and gene regulation atlases have been produced for other model organisms, a relatively limited number of studies have been performed in zebrafish. Given the importance of zebrafish as a model organism for developmental biology, there is a need for detailed atlases at single-cell resolution across multiple developmental stages as well as for moving beyond gene expression to mechanisms of gene regulation. Addressing this challenge, Scott Lacadie and colleagues^1^ report, in this issue of *Cell Genomics*, a single-cell gene regulation atlas of the zebrafish embryo. The authors provide a valuable open resource for the genomics, molecular, and developmental biology communities as well as important insights into the how of gene regulation in zebrafish.

From a mammalian perspective, the first 24 h of a fertilized zebrafish egg’s life is a hyper-accelerated version of vertebrate development. In the first 3 h, the embryo goes from a single cell to approximately 1,000 cells, and by the end of the first 24 h, the embryo has a head, a tail, and many rudimentary organs. In contrast, a human embryo will go through roughly one cell division in that same 24-h time frame. That initial flurry of cell division in the zebrafish embryo is driven by maternally supplied proteins and mRNAs that are packed into the egg before fertilization, but after that initial burst, a massive shift occurs during what is known as the “maternal to zygotic transition” or MZT. During the MZT, the maternal messages are targeted for degradation,^2^ and mRNA transcription from the genome begins in earnest. It is also around this time that we start to see the emergence of specific regions of the embryo that are expressing different sets of genes, a key moment in bilaterian development.

A particularly elegant examination of this phenomenon used single-cell transcriptional profiling at early time points during zebrafish development.^3^ From these data, they were able to perform a three-dimensional reconstruction of gene expression in the early embryo based on single-cell transcriptomics data and integrating that data with known expression patterns from *in situ* hybridizations to characterize cell lineages during this dynamic phase of zebrafish development. However, careful documentation of gene expression at the individual cell level is not the full story. Although we are starting to learn which genes are activated (and repressed) and, to a lesser extent, where they are activated, in order to fully understand this process, we also need to know *how* the genes are turned on or off in each region of the embryo.

In this issue of *Cell Genomics*, McGarvey et al.^1^ get us a step closer to the *how*. The authors used a single-cell version of the chromatin accessibility assay ATAC sequencing termed “sci-ATAC-seq”^4^ to identify potential *cis* regulatory elements in a 24-h-old zebrafish embryo. Data were collected from 23,000 individual cells, and sequence data depth was more than 10,000 reads per cell. Originally, ATAC-seq aggregated fragmented DNA from thousands of cells to identify chromatin accessible to the transposase reaction; however, at single-cell resolution, the number of integration events efficiently captured from even 10,000 fragments is relatively sparse data in a 1.5 billion base pair genome. This is a common issue faced in single-cell ATAC-seq studies and requires creative approaches to data analysis. Typically, the solution is to aggregate the data from all cells to identify accessible chromatin features (the genomic equivalent of stacking confocal image slices) and then, in an iterative fashion, sub-cluster cells that, at much lower resolution, appear to possess similar feature profiles.^5^

McGarvey et al.^1^ used a different approach in their analyses to characterize the early regulatory network in zebrafish larvae. Adapting a strategy utilized effectively in the ENCODE project^6^ to identify chromatin states, the authors developed a method, based on a hidden Markov model (HMM), named “single-cell regulatory landscape segmentation” or “ScregSeg.” Using ScregSeg, the authors made state predictions for each cell based on transposase events across the entire genome in 1-kb windows and then, from the aggregate data, generating the most probable state for each 1-kb region. For approximately 1.5% of the zebrafish genome, the state could be determined to very high confidence based on a variety of parameters, resulting in 71,550 chromatin features that were used to categorize all the cells. From there, it could be established which cells essentially “comply” with the predicted state for each of these features, and dimensionality reduction and clustering could be performed based on this compliance. One of the potential advantages of this approach in sparse data is that HMM can connect neighboring regions instead of identifying each “peak” individually. This may increase sensitivity for adjacent but weaker signals in single-cell data.

Comparing their data to published single-cell RNA sequencing (scRNA-seq) data of the matching 24-h developmental time point, the authors found that they could robustly assign their clustered cell types to cells defined by mRNA expression.^7^ If they then re-ran the HMM on those 17 clusters, which represent candidate cell types, additional granularity could be detected within each cluster. To further validate their data, the authors performed additional sci-ATAC-seq comparing the wild-type profiling to *cloche* mutant embryos. The formerly enigmatic and difficult to clone *cloche* mutant^8^ has been studied for over two decades because of its dramatic lack of almost all blood and endothelium cells. Comparing *cloche* mutant data to their wild-type siblings showed the expected disappearance of the cell clusters identified as blood and endothelium while also showing a surprising increase in the number of cells in clusters identified as muscle or epithelium. These are the types of observations that are difficult to make using bulk approaches, showing the value of this single-cell resolution resource in providing insights into how transcription factors can influence cell fates.

The identification and categorization of these potential *cis*-regulatory elements (CREs) across multiple zebrafish larval cell types will have immediate significant value to many researchers interested in how gene regulation is achieved in a developing vertebrate. The longer-term potential effect on developmental biology of single-cell chromatin accessibility data in the early embryo, such as that collected by McGarvey et al.,^1^ lies in integrating the identification of enhancer elements with the other single-cell technologies that have recently emerged. For example, in zebrafish early development, we now have approaches that can track cell lineage by CRISPR barcoding,^9^ high-resolution single-cell transcriptional profiling,^10^ and single-cell ATAC-seq (scATAC-seq).^1^

**Figure 1:**
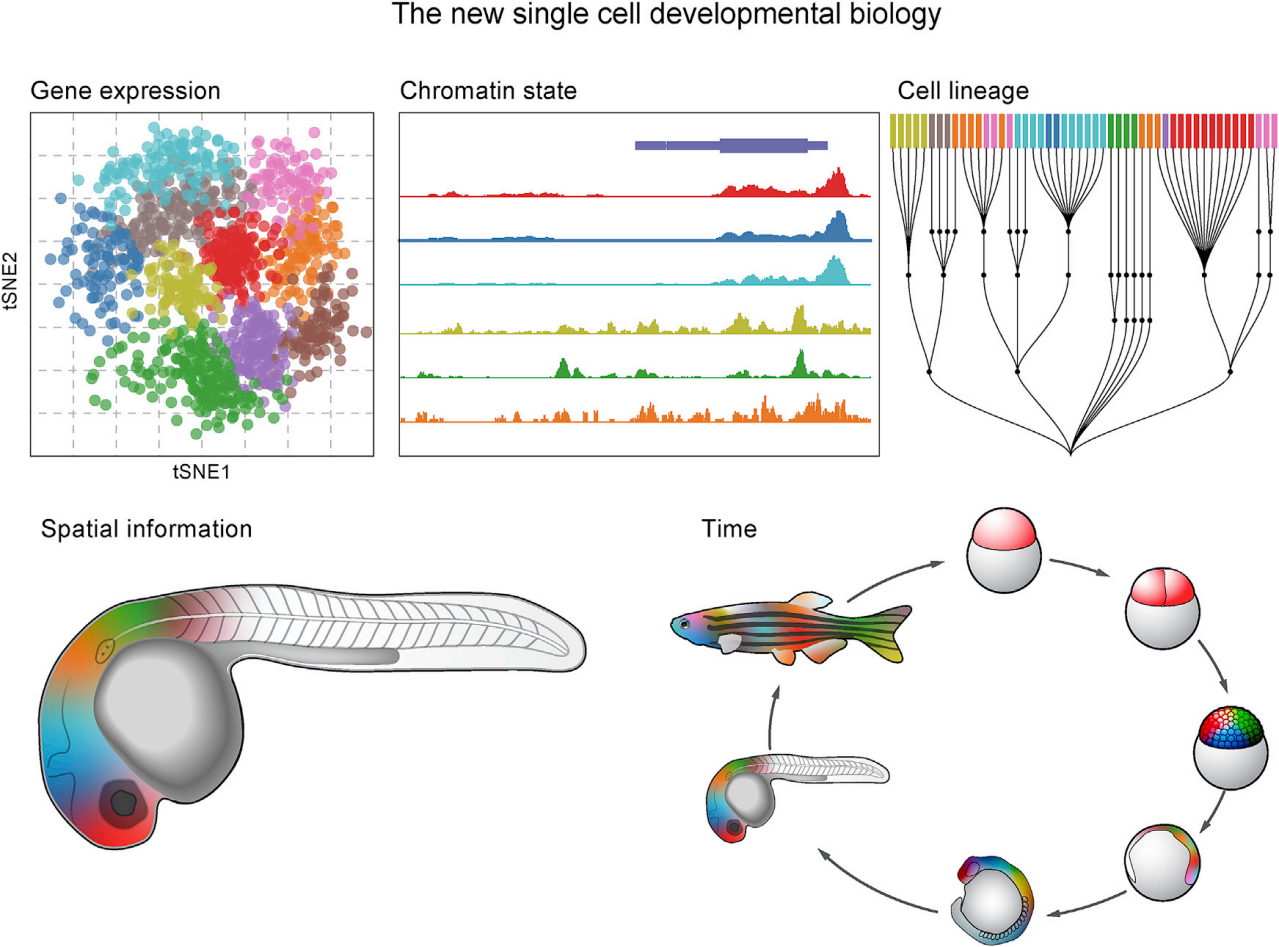
The new single-cell developmental biology The rapid technological development that has allowed many studies to be performed at the resolution of a single cell is improving developmental biologists’ ability to track cell-fate changes at the molecular level. In the near future, a comprehensive and detailed map of early vertebrate development will be possible by integrating multiple approaches that can measured at single-cell resolution: (1) gene expression, (2) chromatin state or accessibility, (3) bona fide cell-lineage tracing, (4) high-resolution spatial expression documentation, and (5) changes in lineage, regulation, and spatial relationships over all stages of development.

The work by McGarvey et al.^1^ is pioneering in characterizing gene regulation in the zebrafish early embryo and providing valuable open resources. Important directions for next studies are to capture together all three technologies (CRISPR barcoding, scRNA-seq, and scATAC-seq) to connect cell lineage, enhancer activation, and subsequent gene activation comprehensively. If those data could also be robustly captured while maintaining true spatial information (instead of imputed based on clustering), the results would provide even deeper insights into the early stages of developmental regulation (Figure 1). With sufficient data depth and spatial reconstruction, it may for the first time be possible in a vertebrate to generate a defined lineage fate map or cell-fate decision tree similar to the one used so beautifully in *C. elegans* research. Developing a cell-fate lineage map for the zebrafish embryo is a worthy goal, much sought after by researchers interested in driving specific cell type differentiation or more broadly in areas such as regenerative medicine.
